# 3*β*-Hydroxy-12-oleanen-27-oic Acid Exerts an Antiproliferative Effect on Human Colon Carcinoma HCT116 Cells via Targeting FDFT1

**DOI:** 10.3390/ijms241915020

**Published:** 2023-10-09

**Authors:** Jue Tu, Xiang Meng, Juanjuan Wang, Ziyi Han, Zuoting Yu, Hongxiang Sun

**Affiliations:** 1 College of Animal Sciences, Zhejiang University, Hangzhou 310058, China; zheyu1114@126.com (J.T.); 13575731715@163.com (X.M.); wjuanjuanhaohao@126.com (J.W.); 15222671162@163.com (Z.H.); 18258972770@163.com (Z.Y.); 2 Laboratory Animal Research Center, Academy of Chinese Medical Sciences, Zhejiang Chinese Medical University, Hangzhou 310053, China

**Keywords:** 3*β*-hydroxy-12-oleanen-27-oic acid (ATA), colon carcinoma cells, antiproliferative, transcriptomics, network pharmacology, FDFT1

## Abstract

3*β*-hydroxy-12-oleanen-27-oic acid (ATA), a cytotoxic oleanane triterpenoid with C14-COOH isolated from the rhizome of *Astilbe chinensis*, has been previously proven to possess antitumor activity and may be a promising antitumor agent. However, its molecular mechanisms of antitumor action were still unclear. This study explored the underlying mechanisms of cytotoxicity and potential target of ATA against human colorectal cancer HCT116 cells via integrative analysis of transcriptomics and network pharmacology in combination with in vitro and in vivo experimental validations. ATA significantly inhibited the proliferation of HCT116 cells in a concentration- and time-dependent manner and induced the cell cycle arrest at the G0/G1 phase, apoptosis, autophagy, and ferroptosis. Transcriptomic analysis manifested that ATA regulated mRNA expression of the genes related to cell proliferation, cell cycle, and cell death in HCT116 cells. The integrated analysis of transcriptomics, network pharmacology, and molecular docking revealed that ATA exerted cytotoxic activity via interactions with FDFT1, PPARA, and PPARG. Furthermore, FDFT1 was verified to be an upstream key target mediating the antiproliferative effect of ATA against HCT116 cells. Of note, ATA remarkably suppressed the growth of HCT116 xenografts in nude mice and displayed an apparent attenuation of FDFT1 in tumor tissues accompanied by the alteration of the biomarkers of autophagy, cell cycle, apoptosis, and ferroptosis. These results demonstrate that ATA exerted in vitro and in vivo antiproliferative effects against HCT116 cells through inducing cell apoptosis, autophagy, and ferroptosis via targeting FDFT1.

## 1. Introduction

Colorectal cancer (CRC) is one of the deadliest diseases globally, ranking third in cancer morbidity and second in cancer mortality worldwide [1]. Despite the benefits of early screening, surgery, and other therapeutic interventions, the five-year relative survival rate of CRC patients has not changed significantly in the past decades [2]. Currently, treatment of colorectal cancer mainly includes surgery, radiotherapy, chemotherapy, and targeted therapies, such as checkpoint inhibitors and anti-angiogenesis therapies [3]; nonetheless, the clinical outcomes are far from being satisfactory due to the considerable side effects and drug resistance. Therefore, there is an urgent need to search for effective and safe agents for CRC prevention and treatment.

**Figure 1:**
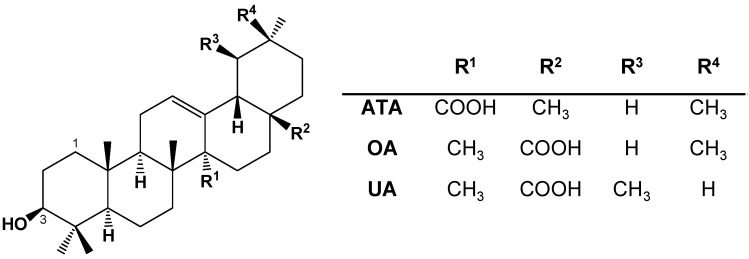
Chemical structures of 3*β*-hydroxy-12-oleanen-27-oic acid (ATA), oleanolic acid (OA), and ursolic acid (UA).

Natural products have been shown to be an excellent and reliable source for the development of new drugs. The plant-derived small-molecule compounds, such as terpenoids, carotenoids, anthocyanidins, and flavonoids, have been extensively investigated for antitumor activities and proven to regulate gene expression and be involved in crucial biological processes such as cell proliferation, differentiation, apoptosis, and autophagy [4]. 3*β*-hydroxy-12-oleanen-27-oic acid (ATA), a cytotoxic oleanane triterpenoid, was isolated from the rhizome of *Astilbe chinensis* (Maxim.) Franch. et Savat. [5]. ATA has the same molecular weight and similar structures to the known antitumor pentacyclic triterpene compounds oleanolic acid (OA) and ursolic acid (UA), with the main difference being the position of the COOH and methyl groups (Figure 1). ATA has been previously reported to exhibit distinctive cytotoxicity towards tumor cells, including human ovarian carcinoma HO-8910 cells, cervical squamous carcinoma HeLa cells, leukemic HL-60 cells, colorectal carcinoma COLO-205 cells, and human hepatoma HepG2 cells [6,7,8], as well as to inhibit the growth of transplanted B16-F10 melanoma in mice [8]. ATA was also reported to be considerably more cytotoxic towards HeLa and COLO-205 cells than its congener OA, implying its favorable structure and superior antitumor effect. In our previous experiment, ATA significantly inhibited the proliferation of a serial of human tumor cell lines, such as bladder carcinoma J82, T24, SW1710, and UM-UC-3, breast cancer MCF-7 and *T47D*, colorectal cancer DLD-1 and HCT15, hepatoma HepG2, non-small cell lung cancer NCI-H460, ovary cancer Caov-3, prostate cancer LNCap (clone FGC), and renal carcinoma Caki-1, as well as normal human umbilical vein endothelial cells (HUVECs) and lung fibroblast MRC-5 cells via the CellTiter-Glo^®^ luminescent cell viability assay (CTG) (Table S1). Notably, the cytotoxic effects of ATA were significantly stronger than its analogue compound OA to all fifteen cell lines. Among them, human colorectal cancer cells DLD-1 and HCT15 were indicated to be more sensitive to ATA than other cells. These results emphasized that ATA might be an excellent antitumor agent, especially for colorectal cancer, and that its unique structure might be responsible for its higher cytotoxic efficiency. Although ATA could inhibit the proliferation of neoplastic cells by inducing apoptosis, more investigations are needed to uncover the in-depth molecular mechanism of ATA to facilitate its development and applications.

High-throughput RNA sequencing (RNA-seq), a promising approach to profile gene expression, has been widely used to unravel molecular mechanisms and explore therapeutic targets of traditional Chinese medicine [9]. Network pharmacology can establish a “compound-target-disease” network by integrating target prediction, statistical analysis network-based algorithms, and biological information analysis, as well as reveal the regulation principles of small molecules in combination with in vivo and vitro experimental validation [10,11]. In the present study, the cytotoxic effects of ATA were first confirmed against a serial of CRC cell lines, which revealed that HCT116 cells were the most sensitive to ATA. Meanwhile, ATA was proved to induce autophagy and ferroptosis in HCT116 cells. Furthermore, the underlying mechanisms of cytotoxic action and the potential targets of ATA were explored via integrative analysis of transcriptomics and network pharmacology in combination with in vitro and in vivo experimental validations.

## 2. Results

### 2.1. ATA Exhibited Cytotoxic Effects towards Colorectal Cancer Cells

**Figure 2:**
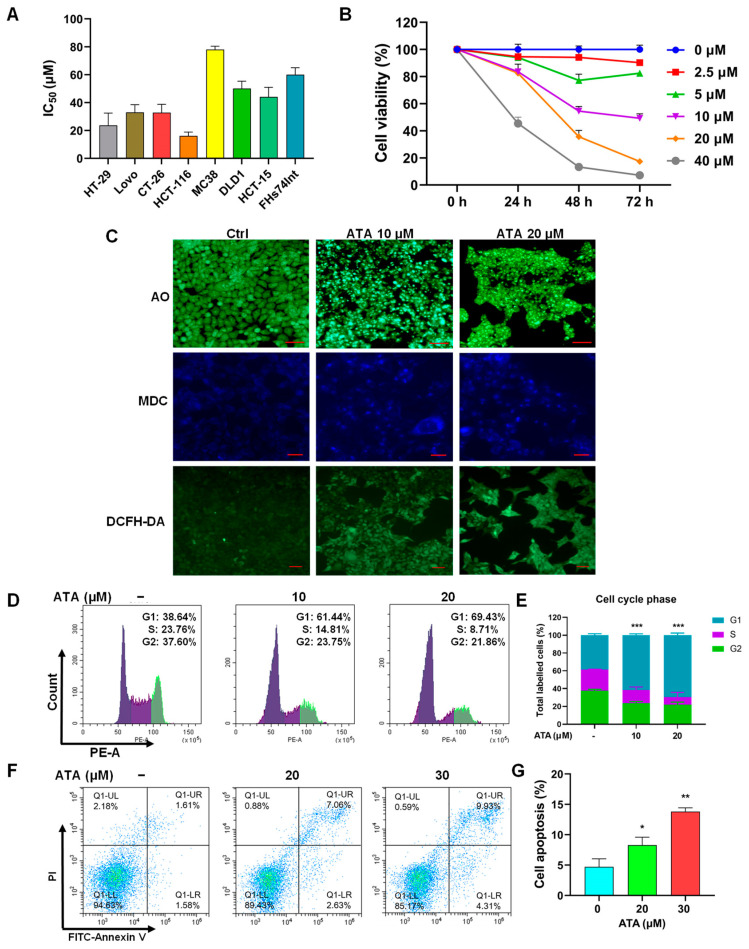
ATA exhibited cytotoxic effects towards colorectal cancer cells. (**A**) The IC_50_ values of ATA towards seven colorectal cancer and human intestinal epithelial FHs74Int cells via the MTT assay. (**B**) The cell viabilities of HCT116 cells treated with ATA at the indicated concentrations for 24 h, 48 h, and 72 h via the MTT assay. (**C**) The morphological changes of ATA-treated HCT116 cells under a fluorescence microscope after incubation with the specific fluorescent dyes AO for apoptosis (up), MDC for autophagy (middle), and DCFH-DA for ROS (below). Scale bars: 20 μm. (**D**,**E**) Cell cycle distribution (**D**) and the percentage (**E**) in various phases of the HCT116 cells treated with ATA (10 μM and 20 μM) for 24 h via FCM. (**F**,**G**) FCM dot plot (**F**) and the apoptotic percentage (**G**) of HCT116 cells after treatment with ATA (20 μM and 30 μM) for 24 h. The figures tagged represent the cell numbers for immediate analysis. Data are expressed as means ± SD for three independent experiments. * *p <* 0.05, ** *p <* 0.01, and *** *p* < 0.001 vs. 0 μg/mL.

The cytotoxic effects of ATA against a series of colorectal cancer cells, including the murine colorectal carcinoma MC38 and CT26, human colon cancer HT-29, Lovo, HCT116, DLD-1, and HCT-15 cell lines, were first investigated using the MTT assay. ATA markedly inhibited the proliferation of all seven colorectal cancer cell lines (Figure 2A). Among them, the most sensitive cell line was HCT116, with its IC_50_ value being 16.17 ± 2.68 μM. ATA significantly inhibited the proliferation of HCT116 in a concentration- and time-dependent manner (Figure 2B).

Meanwhile, the morphology of HCT116 cells treated with ATA for 24 h was also observed under a fluorescence microscope. After AO staining, the DNA in the nuclei of control cells homogeneously exhibited *Kelly* fluorescence, while the ATA-treated cells showed typical apoptotic features characterized by volume reduction, chromatin condensation, nuclear fragmentation with a dense *Kelly* fluorescence stain, and appearance of apoptotic bodies (Figure 2C). MDC is an auto-fluorescent compound reported to specifically label autophagic vacuoles. The control cells exhibited diffused MDC-labeled fluorescence, while the ATA-treated cells demonstrated a punctate pattern of MDC-labeled fluorescence (Figure 2C), suggesting that ATA induced autophagy in HCT116 cells in a concentration-dependent manner. Ferroptosis, a newly identified form of oxidative cell death that is genetically, biochemically, and morphologically distinct from necrosis, autophagy, and apoptosis [12], has been recently reported to be involved in CRC progression [13], even emerging as a therapeutic strategy to trigger KRAS mutant CRC cell death [14]. Therefore, the intracellular ROS level, an indirect hallmark of ferroptosis [15], was detected using DCFH-DA. ATA notably increased intracellular ROS levels represented via the fluorescence intensity of DCF (Figure 2C).

To verify the morphological changes of HCT116 cells exposed to ATA for 24 h, the cell cycle transit and apoptosis were detected using FCM. ATA (10 μM and 20 μM) induced a significant cell cycle arrest at the G0/G1 phase (Figure 2D,E). ATA also triggered cell apoptosis in a concentration-dependent manner, with its apoptotic rate being 8.3% and 13.8% for 20 μM and 30 μM, respectively (Figure 2F,G). The above observations indicated that ATA inhibited the proliferation of HCT116 cells through inducing apoptosis, autophagy, and ferroptosis.

### 2.2. Gene Expression Profiling in HCT116 Cells Induced via ATA

**Figure 3:**
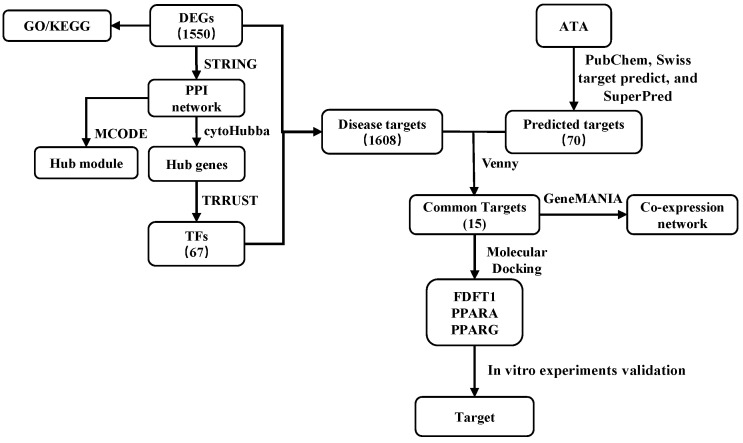
Workflow of the transcriptomic analysis of HCT116 cells treated with ATA.

To clarify the molecular mechanisms of antitumor action of ATA against colorectal cancer, the HCT116 cells treated with ATA (20 μM) for different times were subjected to RNA-seq analysis. The workflow of the transcriptomic analysis is shown in Figure 3.

**Figure 4:**
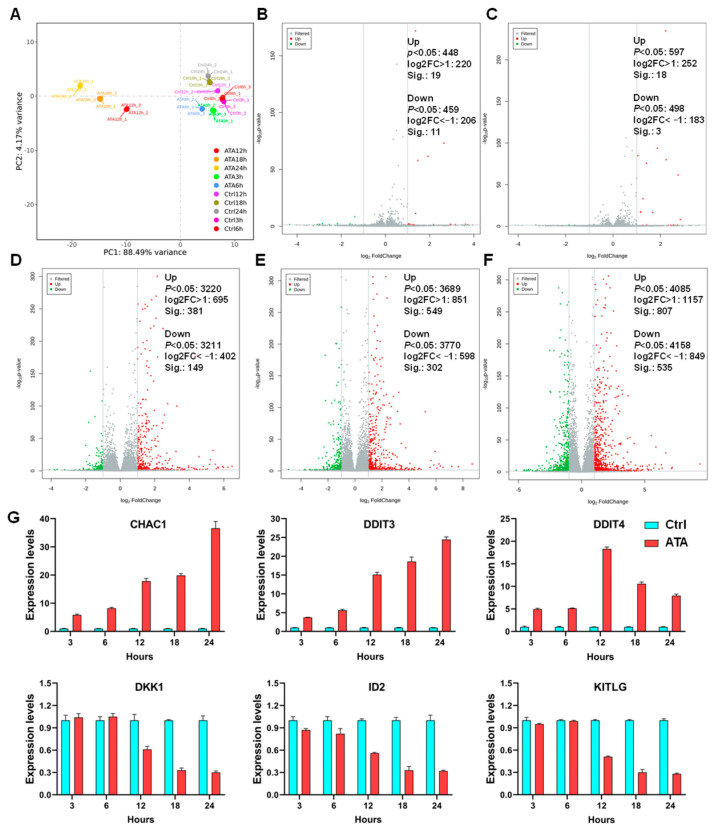
Gene expression profiles in HCT116 cells induced via ATA. (**A**) Principal component analysis (PCA) of the HCT116 cells treated without (Ctrl) or with ATA (20 μM). (**B**–**F**) Volcano plot showing mRNA expression profiles in the HCT116 cells treated without (Ctrl) or with ATA (20 μM) for 3 h (**B**), 6 h (**C**), 12 h (**D**), 18 h (**E**), and 24 h (**F**). Red points represent up-regulation, while green points indicate down-regulation; gray points represent normal expression. (**G**) RT-qPCR validation of 3 up- and 3 down-regulated DEGs.

Principal component analysis (PCA) was first carried out to provide a holistic view of the gene expression profile. As shown in Figure 4A, the groups treated with ATA for 12 h, 18 h, and 24 h were distinctly separated from those for 3 h and 6 h as well as the control group, while samples from each group were clustered. Compared to the untreated control group, only 30 (19 up- and 11 down-regulated) and 21 (18 up- and 3 down-regulated) genes significantly differentially expressed upon ATA exposure for 3 h and 6 h, respectively. However, the cells treated with ATA for 12 h, 18 h, and 24 h showed strong responses at the transcriptome level. The number of differentially expressed genes (DEGs) increased gradually with the prolongation of ATA treatment time (Figure 4B–F). ATA elicited 530 (381 up- and 149 down-regulated), 851 (549 up- and 302 down-regulated), and 1342 (807 up- and 535 down-regulated) DEGs for 12 h, 18 h, and 24 h, respectively (Figure S1). To confirm the validity of the sequencing results, RT-qPCR was undertaken for three putatively up-regulated (CHAC1, DDIT3, and DDIT4) and three down-regulated (DKK1, ID2, and KITLG) genes. The expression levels of the selected genes via RT-qPCR were consistent with the RNA-seq data (Figure 4G and Figure S2), indicating that the result of gene sequencing was reliable.

### 2.3. Function and Hub Genes of DEGs in HCT116 Cells Induced via ATA

**Figure 5:**
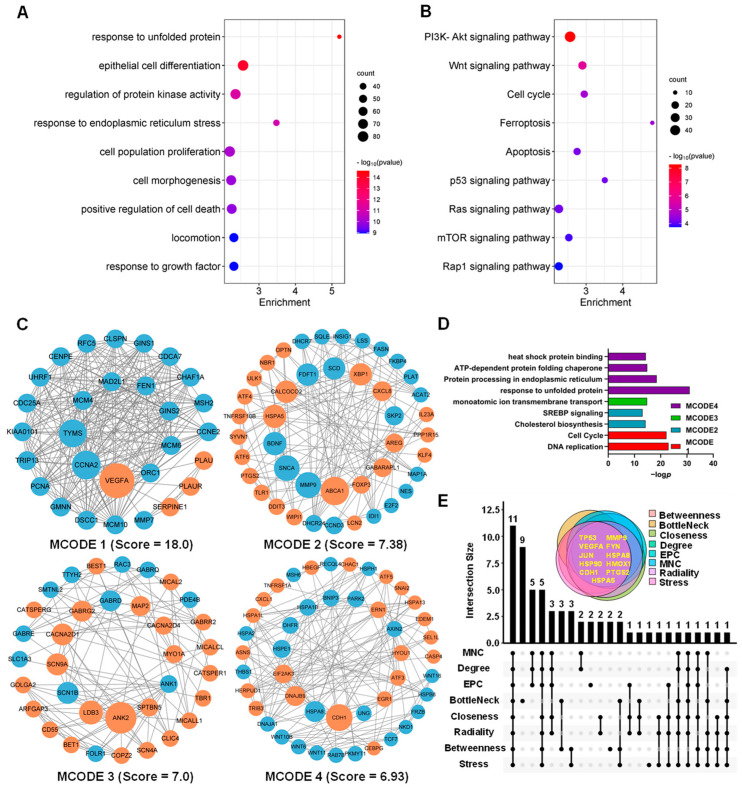
Function and hub genes of the DEGs in HCT116 cells induced via ATA. (**A**,**B**) GO functions (**A**) and KEGG pathways (**B**) of DEGs using Metascape. (**C**,**D**) Four densely connected modules (**C**) and functional annotation (**D**) of the DEGs using the MCODE plug-in of Cytoscape. MCODE 1 contains 29 nodes and 252 edges. MCODE 2 contains 43 nodes and 155 edges. MCODE 3 includes 35 nodes and 119 edges. MCODE 4 includes 46 nodes and 156 edges. Red nodes represent up-regulated genes; blue nodes represent down-regulated genes. The genes were laid out in circles according to the node size depending on the betweenness value, where edges indicate straight associations. (**E**) Upset plot of 11 hub genes using 8 Cytoscape algorithms.

The total 1550 DEGs in HCT116 cells induced via ATA at five time points were subjected to GO function and KEGG pathway enrichment analysis, and the results are shown in Figure 5A,B and Figure S3. The biological functions of the total 1550 DEGs were predominantly connected to “response to unfolded protein (UPR, *p* = 2.50 × 10^−15^)”, “epithelial cell differentiation (*p* = 1.17 × 10^−14^)”, “regulation of protein kinase activity (*p* = 5.44 × 10^−12^)”, “response to endoplasmic reticulum (ER) stress (*p* = 9.28 × 10^−12^”, “cell population proliferation (*p* = 6.32 × 10^−11^)”, “cell morphogenesis (*p* = 1.19 × 10^−10^)”, “positive regulation of cell death (*p* = 1.57 × 10^−10^)”, “Locomotion (*p* = 1.13 × 10^−9^)”, and “response to growth factor (*p* = 1.13 × 10^−9^)” (Figure 5A). The enriched KEGG pathways of these DEGs were “PI3K-Akt signaling (*p* = 5.36 × 10^−9^)”, “Wnt signaling (*p* = 1.69 × 10^−6^)”, “Cell cycle (*p* = 2.24 × 10^−5)^”, “Ferroptosis (*p* = 2.90 × 10^−5^)”, “Apoptosis (*p* = 5.90 × 10^−5^)”, “p53 signaling (*p* = 7.00 × 10^−5^)”, “Ras signaling (*p* = 7.20 × 10^−5^)”, “mTOR signaling (*p* = 1.29 × 10^−4^)”, and “Rap1 signaling (*p* = 1.80 × 10^−4^)” (Figure 5B), which are all associated with cell proliferation and cell death.

Meanwhile, DEGs were subjected to the PPI analysis via STRING and 35 densely connected gene modules were extracted from the network using the MCODE algorithm. Top4 MCODEs were visualized using Cytoscape and subjected to functional annotation via GO enrichment. As shown in Figure 5C,D, in MCODE 1 with the highest score (18.00), the down-regulated genes, such as CCNA2, TYMS, MCM4, GINS2, MAD2L1, and FEN1, were extensively involved in DNA replication and the cell cycle. In MCODE 2 with 43 nodes and 155 edges, the down-regulated genes, such as FASN, FDFT1, and SCD*,* were responsible for cholesterol biosynthesis. In addition, the ER stress-related genes, such as HSPA5, XBP1, ATF6, and DDIT3, as well as the autophagy genes, such as CALCOCO2, ULK1, GABARAPL1, and WIPI1*,* were obviously up-regulated via ATA. Consequently, eight algorithms in the cytoHubba plug-in of Cytoscape were employed to screen the top 20 hub genes of 1550 DEGs in the HCT116 cells induced via ATA. Eleven common hub genes, such as TP53, MMP9, VEGFA, FYN, JUN, HSPA8, HSPA5, HMOX1, CDH1, PTGS2, and HSP90AA1*,* were identified using an upset plot (https://www.omicstudio.cn/tool/43, accessed on 13 March 2023, Figure 5E and Table S2). Furthermore, 67 TFs mediating the expression of these 11 common hub genes were predicted using the TRRUST database (Table S3).

### 2.4. Key Target Achievement Integrated with Network Pharmacology and Molecular Docking

**Figure 6:**
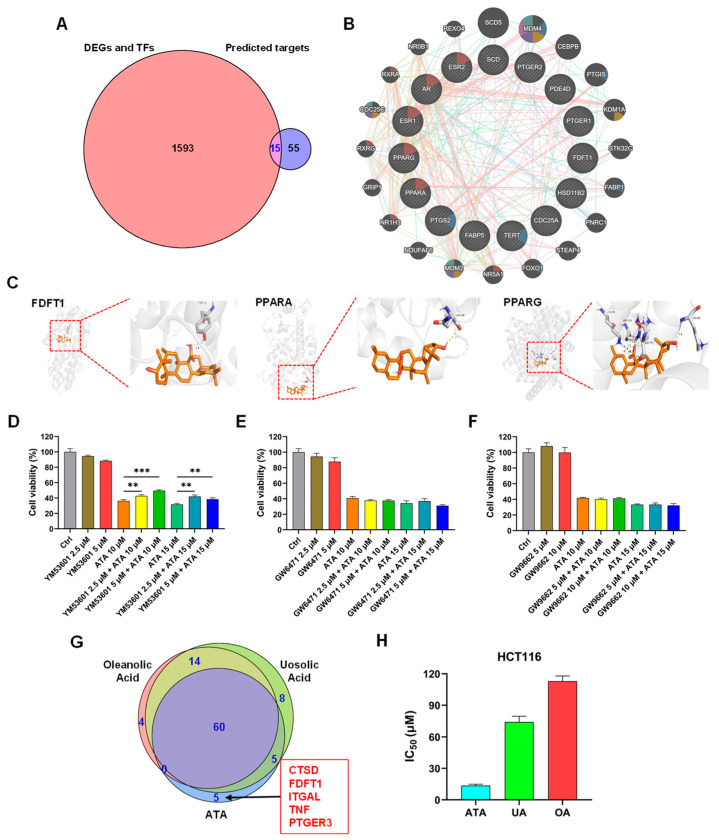
Identification of the antitumor targets of ATA. (**A**) Overlap of the predicted targets of ATA (blue) and disease targets from the transcriptomic data (pink). (**B**) Function analysis of fifteen common targets via GeneMANIA. (C) Binding pattern diagram of ATA and the target proteins FDFT1, PPARA, and PPARG. The yellow dashed line represents the hydrogen bond interaction. (**D**–**F**) After pre-incubation with or without YM-53601 (2.5 μM and 5 µM, (**D**)), GW6471 (2.5 μM and 5 µM, (**E**)), or GW9662 (5 μM and 10 µM, (**F**)) for 30 min, HCT116 cells were treated with ATA (0 μM, 10 μM, and 15 µM) for 48 h. The cell viabilities were detected via the MTT assay. The data are expressed as means ± SD (*n* = 3). ** *p* < 0.01 and *** *p* < 0.001 vs. control (Ctrl). (**G**) Venn diagram of predicted targets for ATA, OA, and UA. Five genes in red were identified as ATA-specific targets. (**H**) The cell viabilities of HCT116 cells treated with ATA, UA, and OA for 48 h were detected via the MTT assay and the IC_50_ values were calculated using Graphpad Prism 9.0 software.

A total of 70 potential targets of ATA were obtained by searching the PubChem, Swiss Target Prediction, and SuperPred databases. A total of 1608 genes were obtained as disease targets by deleting duplicate genes from the 1550 DEGs and 67 predicted TFs in HCT116 cells induced via ATA. The Venn diagram showed that there were 15 common targets, including AR, PPARG, ESR1, PPARA, ESR2, DE4D, PTGER1, PTGS2, CDC25A, FABP5, FDFT1, HSD11B2, PTGER2, SCD, and TERT, between 70 potential targets of ATA and 1608 disease targets (Figure 6A). The co-expression network and related functions of the 15 common target genes were visualized using GeneMANIA. These target genes constructed a complex PPI network with the physical interactions of 43.89%, pathways of 20.98%, shared protein domains of 18.99%, co-expression of 13.15%, co-localization of 2.7%, and genetic interactions of 0.28% (Figure 6B). These fifteen common targets were mainly involved in ligand-activated transcription factor activity, response to oxygen levels, signal transduction in response to DNA damage, signal transduction involved in the mitotic cell cycle checkpoint, G1 DNA damage checkpoint, and mitotic G1/S transition checkpoint, suggesting that ATA inhibited the proliferation of HCT116 cells via these 15 targets. The PPI network of these 15 common targets, with their scores being greater than 0.4, was constructed using Cytoscape, which contained 5 nodes and 7 interaction pairs. PPARG, FDFT1, PPARA, FABP5, and SCD were defined as five key targets using the MCODE plug-in of Cytoscape.

Molecular docking analysis was performed to further identify the direct targets of ATA. The affinities between ATA and the proteins were −8.38, −7.96, −7.91, −7.75, and −7.29 kcal/mol for PPARG, FDFT1, PPARA, FABP5, and SCD, respectively. ATA displayed a close binding conjugation with the protein active site through forming H bonds with the amino acid residues TYR-73 of FDFT1, ASN-299 of PPARA, and LYS-230, ARG-234, ASN-375, and LYS-336 of PPARG (Figure 6C), verifying the reliability of the screened targets based on network pharmacology integrated with the transcriptome and suggesting the key roles of PPARG, FDFT1, and PPARA in mediating the antitumor activity of ATA.

YM-53601, GW6471, and GW9662 were used to specifically inhibit the function of FDFT1, PPARA, and PPARG, respectively. Pretreatment with the FDFT1 inhibitor YM-53601 significantly reversed the decrease in the viability of the HCT116 cells induced via ATA, while both the PPARA inhibitor GW6471 and PPARG inhibitor GW9662 did not affect the cytotoxicity of ATA towards HCT116 cells (Figure 6D–F), indicating that FDFT1 was involved in the cytotoxicity of ATA towards HCT116 cells. More interestingly, FDFT1 was found to be one of ATA’s unique targets, distinct from its two analogs UA and OA, predicted via network pharmacology (Figure 6G and Table S4). ATA, UA, and OA have the same molecular weight and similar chemical structure, with the main difference among them being the position of the COOH and methyl groups (Figure 1), which was structurally responsible for their differences in the cytotoxicity against tumor cell lines. Under the same experimental conditions, the IC_50_ values were 13.7 ± 1.23, 74.10 ± 5.40, and 112.95 ± 5.02 μM towards HCT116 cells for ATA, UA, and OA, respectively (Figure 6H), suggesting that the cytotoxicity of ATA against tumor cells was significantly higher than that of UA and OA. These results suggest that FDFT1 may be the key antitumor target of ATA and is responsible for its distinctive antitumor mechanism different from UA and OA.

### 2.5. FDFT1 Mediated the Cytotoxicity of ATA towards HCT116 Cells

**Figure 7:**
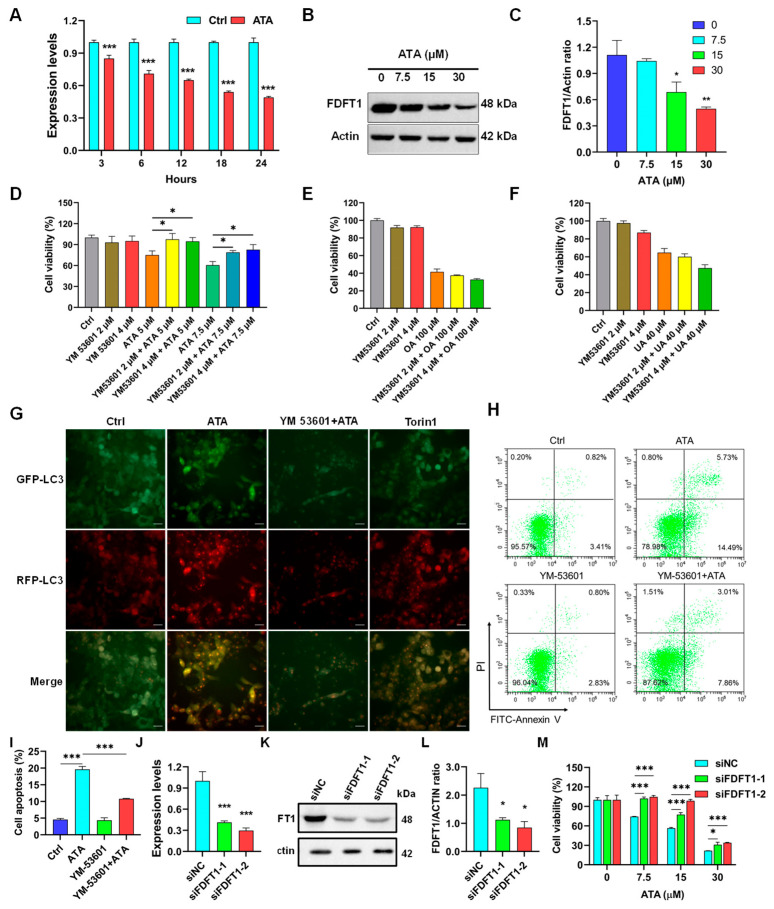
FDFT1 mediated the cytotoxicity of ATA towards HCT116 cells. (**A**) The gene expression levels of FDFT1 in the HCT116 cells treated with ATA for different times via the RT-qPCR assay. (**B**,**C**) The protein expression levels of FDFT1 in the HCT116 cells treated with ATA (7.5, 15 and 30 μM) for 24 h via Western blotting. The figure (**B**) shown is representative of three independent experiments. The data (**C**) are expressed as means ± SD (*n* = 3). * *p* < 0.05 and ** *p* < 0.01 vs. Ctrl (0 μM). (**D**–**F**) After pre-incubation with or without the FDFT1 inhibitor YM-53601 (2 and 4 µM) for 30 min, HCT116 cells were treated with ATA (0 µM, 5 µM, and 7.5 µM, D), OA (100 μM, E), or UA (40 μM, F) for 24 h. The cell viabilities were detected via the MTT assay. The data are expressed as means ± SD (*n* = 3). * *p* < 0.05 vs. the control (Ctrl). (**G**–**H**) After pre-incubation with or without YM-53601 (4 µM, 30 min), the HCT116 cells were treated with ATA (20 μM) for 24 h. The autophagic flux was observed through the mRFP-GFP-LC3 assay. The figure (**G**) shown is representative of three independent experiments. The cell apoptosis was determined using FCM. The figure (**H**) is representative of three independent experiments. The apoptotic percentages (**I**) are expressed as means ± SD (*n* = 3). *** *p <* 0.001 vs. Ctrl. (**J**–**L**) The mRNA (**J**) and protein (**K**,**L**) expression levels of FDFT1 in HCT116 cells were detected using RT-qPCR and Western blotting after transfection with FDFT1 siRNA for 24 h and 48 h, respectively. siNC—negative control siRNA. The figure (**K**) shown is representative of three independent experiments. The data (**J**,**L**) are expressed as means ± SD (*n* = 3). * *p* < 0.05 and *** *p* < 0.001 vs. siNC. (**M**) The HCT116 cells were transfected with FDFT1 siRNAs for 48 h, followed by exposure to ATA at indicated concentrations for another 48 h. The cell viabilities were detected through the MTT assay. The data are expressed as means ± SD (*n* = 3). * *p* < 0.05 and *** *p* < 0.001 vs. siNC.

To validate the key role of FDFT1 in mediating the cytotoxic effect of ATA towards HCT116 cells, the mRNA and protein expression levels of FDFT1 were first analyzed using RT-qPCR and Western blotting, respectively. ATA significantly down-regulated the mRNA (Figure 7A) and protein (Figure 7B,C) expression levels of FDFT1 in HCT116 cells in a time-dependent and concentration-dependent manner, which was consistent with the transcriptomic data (Figure S4). Meanwhile, the effect of ATA on the mRNA and protein expression levels of FDFT1 in the other CRC cell lines, including the HT-29, HCT15, and LOVO cells, revealed that ATA also significantly down-regulated the mRNA and protein expression levels of FDFT1 in these CRC cell lines (Figure S5). Pretreatment with the FDFT1 inhibitor YM-53601 significantly rescued the cell death of HCT116 cells induced via ATA, whereas it had negligible effects on the cytotoxicity of UA and OA (Figure 7D–F). The mRFP-GFP-LC3 assay showed that ATA significantly induced autophagy and promoted autophagic flux in HCT116 cells evidenced via red autophagic puncta (mRFP^+^GFP^−^) accumulation like the positive control mTOR inhibitor Torin1. However, YM-53601 significantly abrogated the autophagic process in HCT116 cells induced via ATA, proved by the weakening of both red (mRFP^+^GFP^-^) and yellow puncta (mRFP^+^GFP^+^) (Figure 7G). Similarly, YM-53601 was obviously able to abate the pro-apoptotic effect of ATA on HCT116 cells (Figure 7H,I). Furthermore, FDFT1 knock-down via siRNA interference in HCT116 cells was conducted to validate it as the main mediator of ATA effects. FDFT1 was confirmed to be effectively knocked down through two of the siRNAs (Figure 7J–L). FDFT1 knock-down also significantly elevated the viability of the HCT116 cells treated with ATA compared to the control siRNA (siNC) (Figure 7M). These findings suggested that FDFT1 might be a key target in mediating the cytotoxicity of ATA towards HCT116 cells.

### 2.6. ATA Regulated FDFT1 to Inhibit the Growth of HCT116 Xenografts in Nude Mice

**Figure 8:**
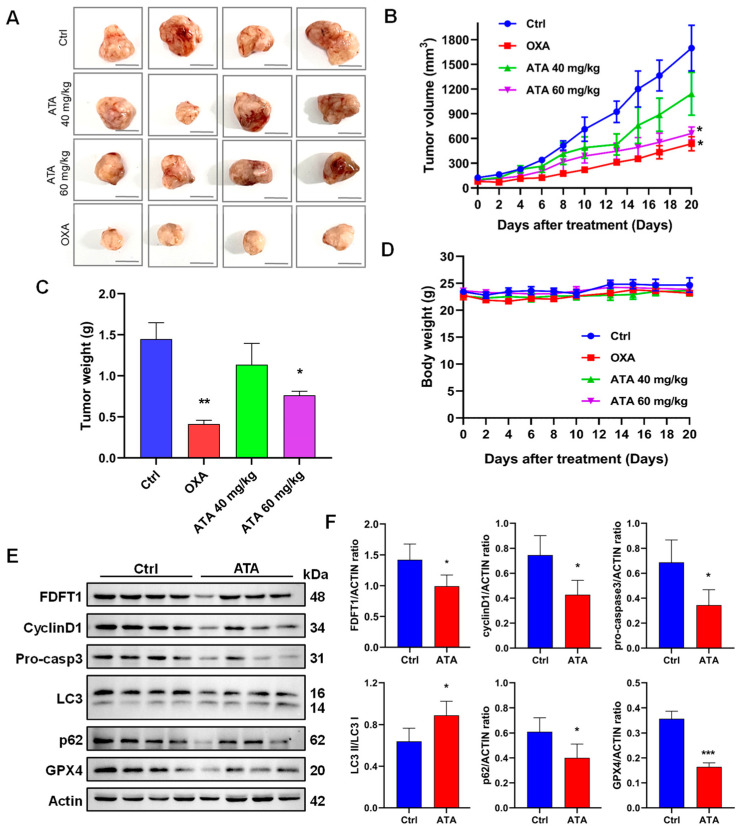
ATA inhibited the tumor growth of HCT116 xenografts in nude mice. (**A**) Tumor anatomy of HCT116 subcutaneous tumor-bearing mice. Scale bar = 10 mm. (**B**) Growth curve of tumor volume. (**C**) Average final tumor weights of each group. (**D**) Body weights of tumor-bearing mice. (**E**,**F**) Expression levels of FDFT1, LC3, p62, cyclinD1, pro-caspase3, and GPX4 in tumor tissues from Ctrl and ATA (60 mg/kg) groups via Western blotting. The data were expressed as means ± SD (*n* = 4). * *p* < 0.05, ** *p* < 0.01, and *** *p* < 0.001 vs. Ctrl.

To validate the in vitro results, the effects of ATA on the tumor growth of HCT116 xenografts in nude mice were determined. ATA significantly reduced xenograft tumor volumes and weight compared with the control group, while the body weights of mice were not affected by ATA (Figure 8A–D). As a positive control, oxaliplatin (Oxa) exhibited a high inhibitory rate on HCT116 xenografts in mice.

Meanwhile, the expression of FDFT1 and marker proteins for the cell cycle, apoptosis, ferroptosis, and autophagy in tumor tissues was detected through Western blotting. ATA noticeably down-regulated the protein expression levels of FDFT1 in tumor tissues of nude mice (Figure 8E,F), and it was found that the expression levels of the G1 phase (cyclin D1)-, apoptosis (pro-caspase3)-, and anti-ferroptosis (GPX4)-related proteins, as well as the autophagy receptor p62 were down-regulated, while those of the autophagy markers LC3 II/I were up-regulated in tumor tissues via ATA (Figure 8E,F), suggesting that ATA resulted in G0/G1 arrest and promoted apoptosis, ferroptosis, and autophagy in xenograft tumors, consistent with the in vitro experiment.

## 3. Discussion

ATA has been previously proven to possess in vitro and in vivo antitumor activities and might be a promising antitumor agent with a unique chemical structure, high efficiency, and low toxicity [5,6,7,8]. In the pre-experiment, the inhibitory effects of ATA on the proliferation of 15 tested cell lines were found to not only be superior to its congener OA, as well as the two other currently widely researched and developed plant-derived compounds 23-hydroxybetulinic acid and *β*-elemene, but equivalent to the positive control drugs cisplatin and oxaliplatin (Table S1). In view of that, human colorectal cancer DLD1 and HCT15 cells seemed to be more sensitive to ATA than other cell lines among the tested tumor cell lines; thus, the cytotoxic effects of ATA against a serial of CRC cell lines were first detected. HCT116 cells were found to be the most sensitive to ATA (Figure 2A). In this study, the antitumor effect of ATA against colorectal cancer and its molecular mechanism were explored via transcriptomic analysis and network pharmacology using HCT116 cells.

ATA significantly suppressed the proliferation of HCT116 cells in a concentration- and time-dependent manner (Figure 2B). Meanwhile, ATA-treated HCT116 cells showed typical morphological changes of apoptosis, autophagy, and ferroptosis after specific fluorescence staining (Figure 2C). FCM analysis also indicated that ATA induces cell cycle arrest at the G0/G1 phase (Figure 2D,E) and apoptosis in HCT116 cells (Figure 2F,G). These initial phenotypic validations of ATA against HCT116 cells were consistent with our previous results [6,7,8], and suggested that ATA might inhibit cell proliferation through inducing cell cycle arrest and various types of cell death involving apoptosis, autophagy, and ferroptosis in CRC cells.

The level of transcription initiation is one of the most pivotal patterns in the regulation of gene expression. Transcriptomic analysis confirmed that ATA-induced DEGs in the HCT116 cells are predominately involved in the cell cycle, ferroptosis, and apoptosis, along with the Wnt and p53 signaling pathways, two well-established drivers of colon cancer (Figure 5B). Since apoptosis resistance is a well-known characteristic of many cancer types, there is a high need for new therapeutic strategies [16]. In this study, it was, for the first time, concerned that ATA induced cell autophagy and ferroptosis, two emerging types of non-apoptotic cell death currently playing important roles in colorectal cancer suppression [17,18], which was further evidenced via the involvement of the typical autophagic mTOR pathway and the positive regulatory PI3K-AKT pathway at the transcriptome level [19]. Moreover, ATA induced endoplasmic reticulum (ER) stress in HCT116 cells, represented via the activation of UPR (Figure 5D), which has been overwhelmingly evidenced to be a critical upstream event for apoptosis [20], autophagy [21], and ferroptosis [22]. Among eleven hub genes, HSPA5, as one of the most important ER chaperones, mediates distinct ER stress signaling cascades [23], while the heat shock proteins HSPA8 and HSP90AA1 are involved in protein refolding and chaperone-mediated autophagy, and HMOX1 takes part in the ferroptosis pathway (Figure 5E). The above results suggested that ATA induces several promising types of cell death through ER stress as the upstream events.

To further identify the key target of ATA for its cytotoxic effects towards HCT116 cells, the integrating analysis of the network pharmacology computational prediction and disease targets from the transcriptomic data was conducted to reveal 15 potential targets of ATA (Figure 6A). These 15 overlapping targets were involved in the oxygen level response, DNA damage, and mitotic cell cycle checkpoint (Figure 6B), suggesting that ATA induces the cell cycle arrest and death of HCT116 cells through regulating these targets. Furthermore, the molecular docking results showed that ATA successfully docked with the three proteins FDFT1, PPARα, and PPARγ (Figure 6C). However, only the FDFT1 inhibitor significantly blocked the cytotoxicity of ATA towards HCT116 cells (Figure 6D–F). More notably, FDFT1 was exactly presented as one of the unique targets for ATA distinct from its two analogs UA and OA (Figure 6G). Although having the same molecular formula and a similar structure, ATA exhibited higher levels of cytotoxic activity on the HCT116 cells than UA and OA (Figure 6H and Table S1). FDFT1 encodes farnesyl-diphosphate farnesyltransferase 1, a membrane-associated enzyme acting at a branch point in the mevalonate pathway and playing an important role in cholesterol biosynthesis [24]. The genes responsible for cholesterol biosynthesis in the ATA-treated HCT116 cells were down-regulated and enriched in the critical module MCODE2 (Figure 5C,D). In this study, ATA significantly down-regulated the mRNA and protein expression levels of FDFT1 in several CRC cell lines, including the HCT116, HT-29, HCT15, and LOVO cells (Figure 7A–C and Figure S5). Moreover, the inhibitory effects of ATA on the mRNA and protein expression levels of FDFT1 in four CRC cell lines were consistent with its cytotoxicity towards these cell lines (Figure 1A). These findings suggested that FDFT1 acting at the beginning of the steroid biosynthesis pathway might be exactly the ATA-specific target biologically responsible for its distinctive antitumor mechanism. However, the inhibitory effects of ATA were not directly related to the basal expression level of FDFT in these cells (Figure S5B), suggesting that FDFT1 might not be the only target of ATA against CRC cell lines.

Recently it has been demonstrated that disordered cholesterol homeostasis is a carcinogenic factor, thus leading to cancer progression [25]. Changes in cholesterol biosynthesis, one of the important manifestations of lipid metabolism reprogramming, are regarded as a hallmark of a variety of cancers and are indispensable for cancer cell survival and proliferation [26]. The abnormal expression of FDFT1 occurs in various cancers, which might be a novel candidate biomarker and a putative new target for cancer therapy [27]. At present, there is little research on the role of FDFT1 in CRC, and the results are also controversial. The gene expression profiling interactive analysis (GEPIA) database (http://gepia.cancer-pku.cn/, accessed on 11 February 2023) revealed that FDFT1 is highly expressed in colon cancer tissues (Figure S6A) and that FDFT1 expression was associated with a better prognosis (*p* = 0.018, log-rank test) (Figure S6B) based on 275 tumor samples and 349 normal tissue samples from TCGA normal and GTEx data. Although it was also reported that high FDFT1 expression levels predicted better prognoses for patients with CRC in the Fudan University Shanghai Cancer Center (FUSCC) cohort (*p* = 0.0238, log-rank test), the expression levels of the FDFT1 gene were significantly lower in CRC tissues than in normal tissues in the GDS2609 and GDS4382 datasets [28]. Luo et al. reported that FDFT1 expression was higher in CRC tissues than normal tissues (*p* < 0.05), and immunohistochemical results also confirmed that FDFT1 was highly expressed in CRC tissues. FDFT1 silencing obviously inhibited the tumor growth in both intraperitoneal implantation and subcutaneous xenograft models of CRC in C57BL/6 mice (*p* < 0.05), but the colorectal peritoneal metastasis model of NCG mice presented no difference in tumor growth after silencing FDFT1 (*p* > 0.05), suggesting that FDFT1 promoted the growth of CRC in vivo with dependence on the tumor immune microenvironment [29].

**Figure 9:**
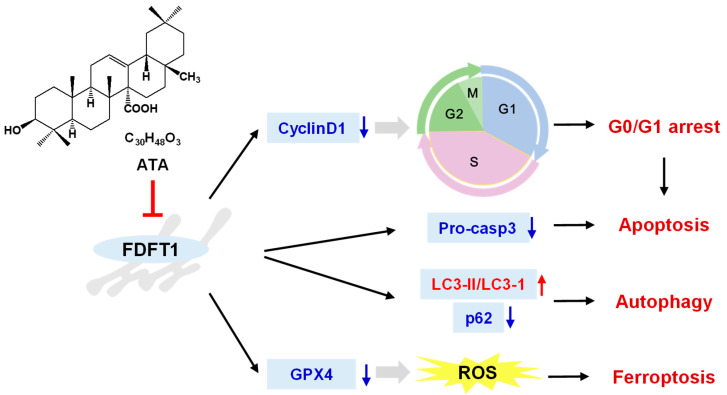
Hypothetical pathway of FDFT1 in mediating the cytotoxic effect of ATA against HCT116 cells.

Recently, FDFT1 has been identified as a ferroptosis-related gene and is considered an important gene for prognosis prediction in colorectal cancer patients [30,31]. Gathering evidence has indicated that FDFT1 expression increases in cells undergoing proliferation, suggesting that FDFT1 is implicated in proliferative signaling in cancer cells. In fact, FDFT1 regulates the cell cycle, where the inhibition of FDFT1 significantly impedes cells in the S-phase. FDFT1 could activate the NF-kB pathways, leading to an increase in the levels of anti-apoptotic proteins, such as Bcl-xL, Bcl-2, and Bax, and a decrease in the levels of pro-apoptotic proteins, such as caspase-3, thereby blocking apoptosis signaling [32]. In addition, the high expression levels of FDFT1 increased the intracellular squalene levels, which protected the cell membrane from lipid peroxidation by reactive oxygen species (ROS) and further prevented the cells from entering the ferroptosis pathway [33]. FDFT1 also participated in the biological process of ferroptosis through the Akt signaling pathway [34], and the inhibition of FDFT1 could increase endogenous geranylgeranoic acid levels, resulting in incomplete autophagy, along with a reduction in cholesterol levels also inducing autophagy [35]. The inhibition assay further confirmed that inhibition of FDFT1 could reverse the effect of ATA in the regulation of cellular proliferation and induction of autophagy and apoptosis (Figure 7D,G–I). Meanwhile, FDFT1 knock-down significantly weakened the anti-proliferative effect of ATA on the HCT116 cells (Figure 7M). More than that, ATA exhibited potent antitumor activity in the HCT116 xenograft mice (Figure 8A–C). Meanwhile, the protein expression levels of FDFT1 was notably down-regulated, accompanied by the alteration of critical biomarkers of the cell cycle, autophagy, apoptosis, and ferroptosis in the tumor tissues from the ATA-treated mice (Figure 8E,F). These results indicated that ATA exerted an antiproliferative effect against the HCT116 cells by targeting FDFT1 as a tumorigenesis gene in the present study (Figure 9).

In this study, ATA significantly down-regulated the mRNA and protein expression levels of FDFT1 in HCT116 cells. Due to the lack of existing test kits, the impact of ATA on FDFT1 enzyme activity has not been tested. However, YM-53601, an inhibitor of FDFT1 enzyme activity [36], significantly blocked the cytotoxicity of ATA against HCT116 cells at a non-cytotoxic concentration. The drugs with the same mechanism of action may exhibit competitive antagonism when combined due to varying intrinsic activities and affinities. From this fact, it was thereby speculated that ATA may also inhibit FDFT1 enzyme activity. Therefore, whether the specific mechanism is due to transcriptional blockade, protein degradation, and/or enzyme activity inhibition remains to be further explored.

This study demonstrated that ATA possessed the cytotoxic activities against HCT116 cells by inducing cell apoptosis, autophagy, and ferroptosis in vitro and in vivo. In mechanisms, ATA exerted the antiproliferative effect against HCT116 cells through targeting FDFT1. These data further expanded current knowledge on the mechanism of antitumor action of triterpenoid compounds and provided a new target for the research and design of anticancer agents.

## 4. Materials and Methods

### 4.1. Materials and Reagents

For this study, 3-(4,5-dimethyl thiazolyl-2)-2,5-diphenyltetrazolium bromide (MTT), acridine orange (AO), and dimethyl sulfoxide (DMSO) were purchased from Sigma-Aldrich, St. Louis, MO, USA; cell culture medium, trypsin, penicillin, streptomycin, and fetal bovine serum (FBS) were obtained from Gibco, Burlington, MA, USA; TRIzol was purchased from Invitrogen, Carlsbad, CA, USA; reverse transcriptase, oligo(dT)_18_, and ribonuclease inhibitor were obtained from Shanghai Sangon Biotech Co., Ltd., Shanghai, China; RIPA lysis buffer, BCA protein assay kit, anti-mouse actin mAbs, anti-caspase3 antibody (AF0081), horseradish peroxidase (HRP)-conjugated goat anti-rabbit and anti-mouse IgG (H + L), enhanced chemiluminescence (ECL) kit, and reactive oxygen species (ROS) assay kit were purchased from Beyotime, Shanghai, China; phosphatase inhibitor and protease inhibitor cocktails were obtained from Biotool, Houston, TX, USA; anti-FDFT1 antibody (ab195046) was purchased from Abcam, Cambridge, UK; anti-cyclinD1 antibody (ET1601-31) was obtained from HUABIO, Shanghai, China; anti-GPX4 (67763-1-Ig) antibody was obtained from Proteintech, Rosemont, IL, USA; the antibodies against LC3A/B (4108S) and p62 (39749S) were purchased from CST, Danvers, MA, USA; a monodansylcadaverine (MDC) sensor kit was obtained from KeyGEN BioTECH Corp., Ltd., Nanjing, China; an annexin V-FITC apoptosis detection kit and propidium iodide (PI)/RNase staining buffer were obtained from BD Pharmingen, San Diego, CA, USA; mRFP-GFP-LC3 lentivirus was obtained from Genechem, Shanghai, China.

3*β*-Hydroxy-12-oleanen-27-oic acid (ATA, C_30_H_48_O_3_, MW: 456.3594, Figure 1) was previously isolated from the rhizomes of *A. chinensis* [5]. The purity of ATA was determined to be 98.9% through HPLC. Ursolic acid (UA, ≥ 98%), oleanolic acid (OA, ≥ 98%), and 23-hydroxybetulinic acid (23-HA, ≥ 98%) were purchased from Chengdu Lemeitian Pharmaceutical Technology Co., Ltd., Sichuan, China; *β*-elemene (≥ 98%) was obtained from Shanghai Yuanye Bio-Technology Co., Ltd., Shanghai, China; cisplatin, oxaliplatin, PPARγ inhibitor GW9662, and PPARα inhibitor GW6471 were purchased from Selleck Chemicals, Houston, TX, USA; the FDFT1 inhibitor YM-53601 was obtained from MedChemExpress Technology, Monmouth Junction, NJ, USA. The stock solutions in DMSO were prepared and diluted as desired with the cell culture medium. The final concentration of DMSO in all experiments was less than 0.1% and did not show any detectable effect on cell growth or apoptosis.

### 4.2. Cell Lines and Culture

Murine colon carcinoma CT26, human bladder carcinoma J82, T24, SW1710, and UM-UC-3, breast cancer MCF-7 and T47D*,* colorectal cancer DLD-1, HCT15, HT29, Lovo, and HCT116, hepatoma HepG2, non-small cell lung cancer NCI-H460, ovary cancer Caov-3, prostate cancer LNCap clone FGC, and renal carcinoma Caki-1, as well as normal HUVEC, intestinal epithelial FHs74Int, and lung fibroblast MRC-5 cell lines were obtained from the National Collection of Authenticated Cell Cultures. Murine colorectal adenocarcinoma MC38 cell lines were purchased from MingzhouBio, Ningbo, Zhejiang, China. The cells were maintained in the logarithmic phase of growth in DMEM, MEM, McCoy’5a, or RPMI medium supplemented with 10% heat-inactivated FBS, 100 IU/mL penicillin, and 100 μg/mL streptomycin in an incubator at 37 °C with a humidified atmosphere of 5% CO_2_. Cell line identity was validated through short tandem repeat profiling, and routine mycoplasma testing was negative for contamination.

### 4.3. Experimental Animals

BALB/c-nu nude mice (male, five weeks of age) were purchased from Sino-British SIPPR/BK Laboratory Animal Co., Ltd. (Shanghai, China) and housed in a specific pathogen-free (SPF) environment at the Laboratory Animal Research Center of Zhejiang Chinese Medical University. All animal experiments were performed according to the procedures approved by the Institutional Animal Care and Use Committee of Zhejiang Chinese Medical University.

### 4.4. Cell Viability Assay

HCT116 cells were seeded at 1.0 × 10^4^ cells/well in a 96-well plate and incubated at 37 °C in a humidified atmosphere with 5% CO_2_. After 24 h, various concentrations of the tested samples were added into each well, and then cells were incubated for an indicated time. For the inhibition assay, HCT116 cells were pre-treated with YM-53601, GW9662, or GW6471 for 30 min, followed by co-incubation with the tested compounds for an additional 48 h. Each concentration was repeated for four wells. Four hours before the end, the extent of cell proliferation was detected using the MTT assay, as previously described [37].

### 4.5. Cell Morphological Observation

The HCT116 cells were seeded at 1 × 10^5^ cells/mL into a 24-well plate and incubated at 37 °C in a humidified atmosphere with 5% CO_2_. After 24 h, the cells were either treated with ATA (10 μM and 20 μM) or MEM medium for 24 h. For cell apoptotic observation, cells were stained with AO (5 μM) in the culture medium at room temperature for 15 min. For cell autophagy detection, the cells were exposed to MDC (50 μM) at 37 °C for 30 min in the dark for staining using the KEYGEN MDC Sensor kit. The intracellular ROS levels were detected using the ROS assay kit [37]. The cells were incubated with 10 μM 2′,7′-dichlorofluorescein diacetate (DCFH-DA) at 37 °C for 20 min. The stained cells were visualized via fluorescence microscopy (Axiover200, Zeiss, Oberkochen, Germany).

### 4.6. siRNA Transfection

FDFT1 siRNA and negative control siRNA were synthesized by GenePharma (Shanghai, China) and were transfected into HCT116 cells for 24 h or 48 h using Lipofectamine 3000 (Invitrogen, L3000015), according to the manufacturer’s protocol. The siRNA duplexes targeting FDFT1 indicated: siFDFT1-1: 5′-GUGCCUGAAUGAACUUAUATT-3′; siFDFT1-2: 5′-CCAUCUACCUGUCGUUUGUTT-3′.

### 4.7. Real-Time Quantitative Polymerase Chain Reaction (RT-qPCR)

The HCT116 cells were seeded at 1 × 10^5^ cells/mL into a 24-well plate and incubated at 37 °C in a humid air with 5% CO_2_. After 24 h, the cells were either treated with ATA (20 μM) or MEM medium for 3 h, 6 h, 12 h, 18 h, and 24 h. The total RNA was isolated with TRIzol reagent and reverse transcription was performed. The PCR was conducted on a Bio-Rad CFX 96 system using the SYBR Green qPCR Master Mix. The specific primers for RT-qPCR were synthesized by Sangon Biotech Co., Ltd. (Shanghai, China) and the sequences are listed in Table S5. Primer amplification efficiency and specificity were verified for each set of primers. The mRNA expression levels of the tested genes relative to GAPDH were determined using the 2^-ΔΔCt^ method.

### 4.8. Flow Cytometry (FCM)

The HCT116 cells were seeded at 2 × 10^5^ cells/well in 6-well plates and then cultured at 37 °C in a humidified atmosphere with 5% CO_2_ for 24 h. After treatment with ATA for 24 h, the cells were harvested and washed twice with PBS. For cell cycle detection, the collected cells were fixed in ice-cold 70% ethanol for overnight at −20 °C and then centrifugated. The pellet was resuspended in 0.5 mL of PI/RNase staining buffer and incubated for 20 min at room temperature in the dark. For the apoptosis assay, the cells were stained with the Annexin V-FITC/PI Apoptosis Kit according to the instructions. The analysis was performed on the flow cytometer (CytoFLEX, Beckman Coulter, CA, USA) using CytExpert 2.4 software.

### 4.9. RNA Sequencing (RNA-Seq)

The HCT116 cells treated with and without ATA (20 μM) for 3, 6, 12, 18, and 24 h were subjected to RNA-seq. The total RNA was isolated with TRIzol reagent. RNA quality was assessed using the NanoDrop UV–VIS spectrophotometer and Agilent 2100 Bioanalyzer. After digestion with DNase to remove the genomic DNA, mRNA purification and fragmentation were performed using the TruSeq Stranded mRNA LT Sample Prep Kit. The fragmented RNAs were used as templates to synthesize cDNA with reverse transcriptase. The double-stranded cDNA was purified and enriched using the Agencourt AMPure XP kit. Sequencing was conducted using an Illumina HiSeqTM2500 sequencer to afford paired-end data of 125 bp in length after constructing the library. Trimmomatic software was used to control the raw data quality, remove the linkers, and obtain high-quality clean reads [38]. The obtained clean reads were matched to the human genome (GRCh38) using HISAT2to afford bam files [39]. The fragments per kilobase of exon per million reads (FPKM) were quantified using cufflinks software (version 0.16) [40,41].

### 4.10. Differentially Expressed Genes (DEGs) Analysis

Significant difference analysis between samples was calculated using the DESeq R package. Differentially expressed genes (DEGs) were screened based on *p* < 0.05 and fold change (FC) > 2 or < 0.5 compared with control samples calculated on the three replicates. The resulting significance scores were corrected for multiple testing using the Benjamini–Hochberg correction. Principal component analysis (PCA) was carried out to assess the variability and repeatability of samples using the normalized RNA-seq counts. A volcano plot was generated with the average FC and *p*-value using online software (http://sangerbox.com/, accessed on 10 March 2023). A Venn diagram was produced using online software (http://bioinformatics.psb.ugent.be/webtools/Venn/, accessed on 13 March 2023). The heat maps were plotted using https://www.omicstudio.cn/tool (accessed on 12 March 2023). The Gene Ontology (GO) and Kyoto Encyclopedia of Genes and Genomes (KEGG) enrichment analysis were performed using Metascape software (http://metascape.org/, accessed on 11 March 2023) [42].

The protein–protein interaction (PPI) network of DEGs was constructed using the Search Tool for the Retrieval of Interacting Genes (STRING) online database (version 11.5) (https://cn.string-db.org/, accessed on 12 March 2023), and then was visualized using Cytoscape software (v3.9.1) [43,44]. The plug-in molecular complex detection technology (MCODE) in Cytoscape software was used to analyze pivotal functional modules by the following criteria: K-core = 2, degree cutoff = 2, max depth = 100, and node score cutoff = 0.2 [45]. The hub genes were identified using the cytoHubba plug-in of Cytoscape software with eight common algorithms (betweenness, bottleneck, MNC, degree, closeness, stress, EPC, and radiality) [46]. The intersecting genes of the top twenty genes from all eight approaches of CytoHubba were considered as hub genes. The upstream transcription factors (TFs) of the hub genes were predicted using the Transcriptional Regulatory Relationships Unraveled by Sentence-based Text mining (TRRUST) database (https://www.grnpedia.org/trrust/, accessed on 13 March 2023), and an adjusted *p*-value < 0.05 was considered significant [47].

### 4.11. Network Pharmacological Analysis

The structural information of ATA, OA, and UA were downloaded from the PubChem Database (https://pubchem.ncbi.nlm.nih.gov/, accessed on 13 October 2022). The PubChem, Swiss target prediction (http://www.swisstargetprediction.ch/, accessed on 13 October 2022), and SuperPred databases (https://prediction.charite.de/s, accessed on 13 October 2022) were used to predict the potential targets of ATA, OA, and UA. DEGs and TFs regulating the hub genes from transcriptomic analysis were merged as disease targets of ATA. Then, the OmicStudio Venn tool (https://www.omicstudio.cn/tool/6, accessed on 13 March 2023) was applied to produce Venn diagrams and derive the cross genes between the predicted targets and disease targets, which were considered as the candidate targets of ATA. Subsequently, a co-expression network of these key targets was constructed using GeneMANIA (http://www.genemania.org/, accessed on 13 March 2023.) [48]. Further, the unique genes of the predicted targets of the three compounds were respectively obtained using Venn diagrams.

### 4.12. Molecular Docking

The molecular docking simulation was performed using AutoDockTools (4.2.3) to verify the credibility of the hub targets, as previously described. The mechanical structure of ATA was optimized via Chem3D (21.0). The 3D structures of the proteins were obtained from the PDB database (http://www.rcsb.org/pdb/home/home.do, accessed on 2 April 2023). The ligand–receptor binding property was analyzed. Binding energies < −7.0 kcal/mol denoted the docking, and the results were visualized using Pymol software (v2.5).

### 4.13. Western Blot Analysis

The HCT116 cells were seeded at 1 × 10^6^ cells into a 6 cm dish and then incubated at 37 °C for 24 h in a humidified atmosphere with 5% CO_2_. After being treated with ATA for 24 h, the cells were washed twice with cold PBS. The tumor tissues (ca. 75 mg) or the collected cell pellets were lyzed with RIPA lysis buffer containing PMSF using the KZ-II high speed tissue grinder (Servicebio, Wuhan, China). The supernatants were collected through centrifugation at 12,000 rpm for 10 min at 4 °C. The protein concentrations in the supernatants were detected using the BCA protein assay kit. The denatured proteins were separated on a 10% SDS-polyacrylamide gel via electrophoresis and transferred to a polyvinylidene difluoride (PVDF) membrane. After blocking the membrane with 5% skim milk in Tris-buffered saline containing 0.1% Tween-20 (TBST) for 1 h at 37 °C, the blot was incubated with the primary antibodies overnight at 4 °C. Subsequently, the membranes were washed with TBST and incubated with HRP-conjugated goat anti-rabbit IgG (H + L) for 1 h. After washing the membrane with TBST for three times, the signal was visualized with the ECL kit on the iBright™ CL1500 Imaging System.

### 4.14. The mRFP-GFP-LC3 Assay

The mRFP and GFP expressed in mRFP-GFP-LC3 tandem fluorescent protein lentivirus were used to track LC3 [49,50]. The HCT116 cells were plated in confocal dishes and incubated overnight. After being transfected with mRFP-GFP-LC3 lentivirus for 48 h, the cells were selected with 2 μg/mL puromycin for 48 h. The cells stably expressing mRFP-GFP-LC3 were seeded into 6-well plates at a density of 2 × 10^5^ cells in 2 mL of serum-containing medium. After 24 h, the cells were treated with ATA (20 μM) in the presence or absence of YM-53601 (4 μM), or with Torin (1 μM) for 24 h. The cells were visualized using fluorescence microscopy (Axiover200, Zeiss, Germany).

### 4.15. Xenograft Model in Nude Mice

HCT116 cells (1 × 10^7^ cells in 200 μL of PBS) were injected subcutaneously into the flanks of the mice. When the tumor volume reached 80−100 mm^3^ on average, the mice were randomly divided into four groups, each consisting of four mice. The mice were orally administered with ATA at the doses of 40 mg/kg and 60 mg/kg once daily or injected i.p. with oxaliplatin (Oxa) at a dose of 6 mg/kg in saline twice a week for three weeks. The dose volume was 0.2 mL/10 g body weight. The model control (MC) groups received the same volume of saline. Body weights and tumor sizes were measured every two days. Tumor size was measured using a digital slide caliper and volumes (mm^3^) were calculated as follows: Tumor volume (mm^3^) = L × W^2^/2, where L is the length of the tumor and W is the width of the tumor. Animals were sacrificed after the final drug administration and tumors were carefully excised and weighed. The tumor tissues were snap frozen in liquid nitrogen and stored at −80 °C for subsequent analysis.

### 4.16. Statistical Analysis

Data were presented as mean ± SD and examined for their statistically significant differences with the analysis of variance (ANOVA) and Student’s *t*-test. The *p*-values of less than 0.05 were statistically significant. The calculations and graphs were performed using GraphPad Prism 9.0 software (GraphPad Software, San Diego, CA, USA).

## Data Availability

All data are available in the Supplementary Materials.

## Supplementary Materials

The following supporting information can be downloaded at: https://www.mdpi.com/article/10.3390/ijms241915020/s1.
