# Supplementary material for: 3β-Hydroxy-12-oleanen-27-oic Acid Exerts an Antiproliferative Effect on Human Colon Carcinoma HCT116 Cells via Targeting FDFT1
Source: Int J Mol Sci. 2023 Oct 9;24(19):15020. doi: 10.3390/ijms241915020 (PMC10573780; doi:10.3390/ijms241915020)
Supplement: Supplementary file 1 [file ijms-24-15020-s001.zip › 2023-9-30 Supplementary Materials.pdf]

Supplementary Materials

3β-Hydroxy-12-oleanen-27-oic acid exerts the antiproliferative effect on human colon carcinoma HCT116 cells via targeting FDFT1

Jue Tu [1,2], Xiang Meng [1], Juanjuan Wang [1], Ziyi Han [1], Zuoting Yu [1], Hongxiang Sun [1,*]

[1] College of Animal Sciences, Zhejiang University, Hangzhou 310058, China; zheyu1114@126.com (J.T.); 1154026924@qq.com (X.M.); wjuanjuanhaohao@126.com (J.J.W.); 15222671162@163.com (Z.Y. H.); 18258972770@163.com (Z.T.Y.); sunhx@zju.edu.cn (H.X.S.)

[2] *Laboratory Animal Research Center, Academy of Chinese Medical Sciences, Zhejiang Chinese Medical University, Hangzhou, 310053, China*; zheyu1114@126.com (J.T.)

* Correspondence: sunhx@zju.edu.cn; Tel.: +86-570-8898-2091

Supplementary Figures

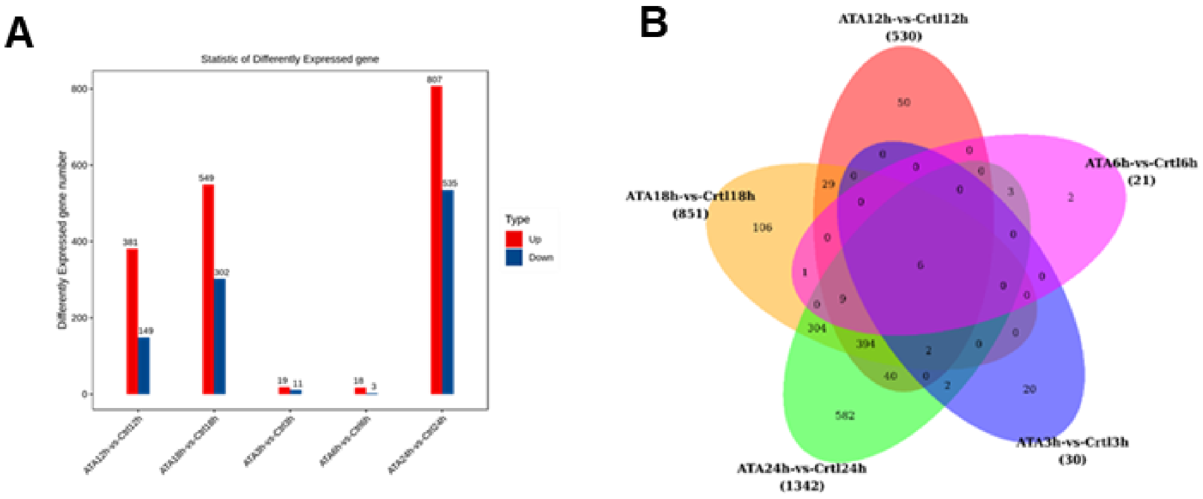

Figure S1. The numbers of up-regulated and down-regulated genes in HCT16 cells induced by ATA at indicated time points.

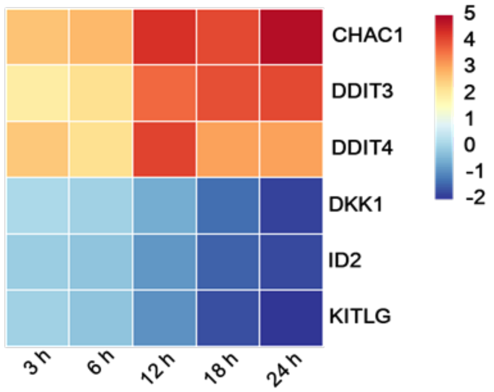

Figure S2. Expression heatmap of the validated genes.

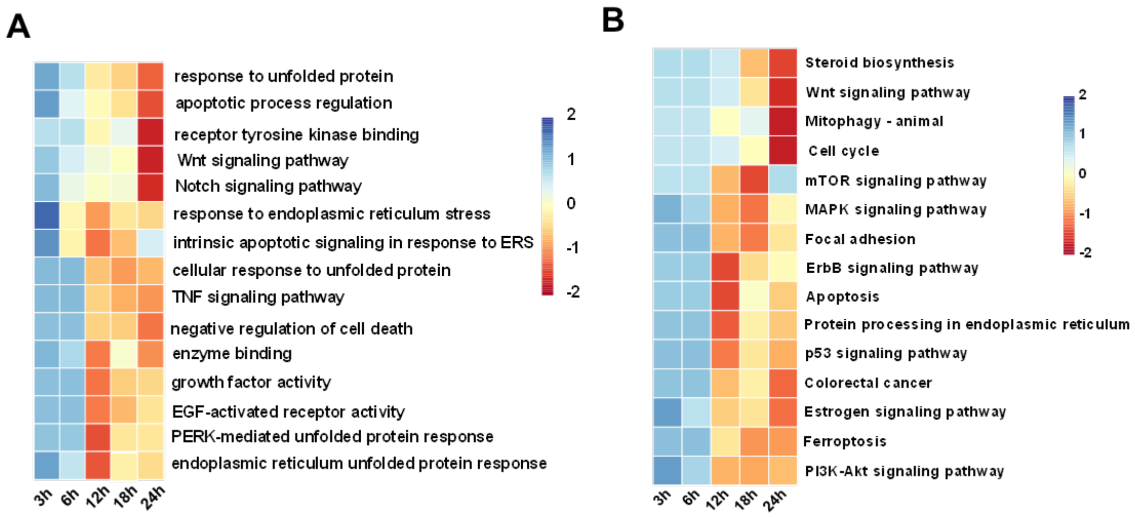

Figure S3. Heatmap of GO function (A) and KEGG pathway (B) enrichment of DEGs in HCT116 cells treated with ATA for different times.

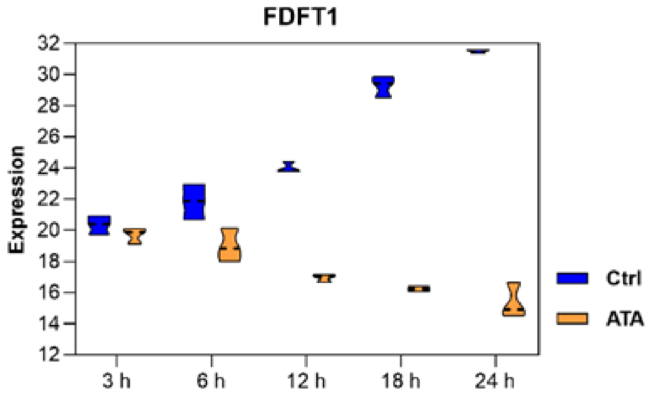

Figure S4. The expression levels of FDFT1 in HCT116 cells treated with ATA by RNA-Seq.

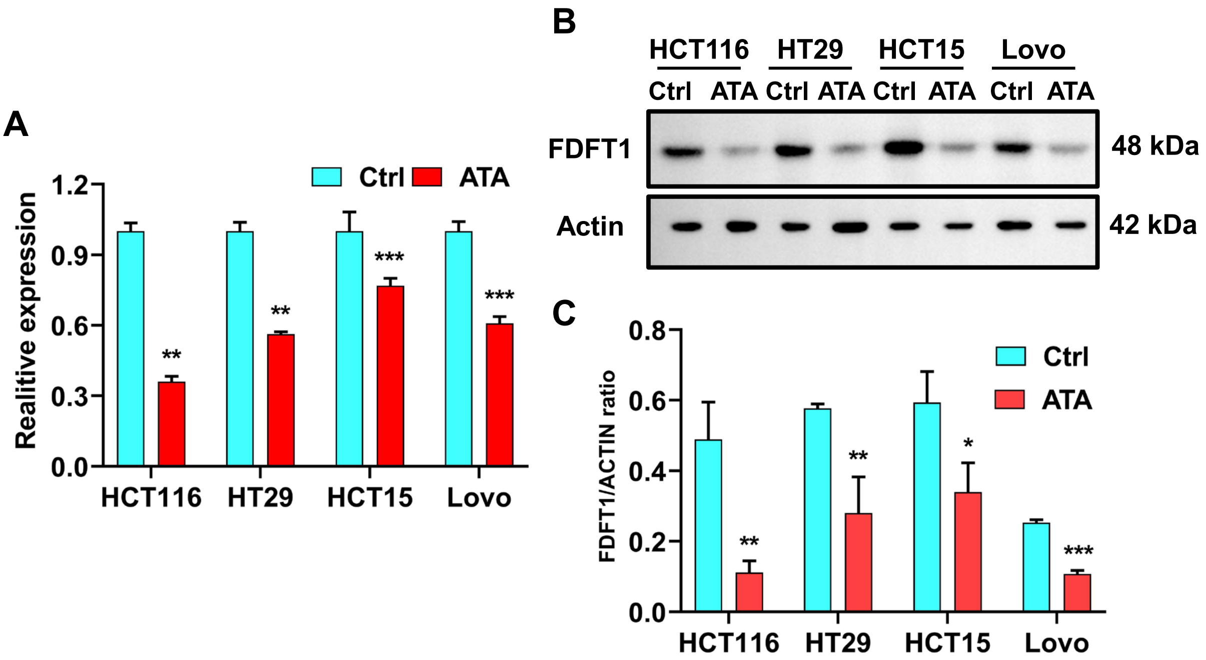

Figure S5. The gene and protein expression of FDFT1 in a diversity of CRC cell lines treated with ATA. (**A**) The gene expression of FDFT1 in HCT116, HT29, HCT15 and Lovo cells treated with ATA (30 μM) for 12 h by RT-qPCR assay. (**B**,**C**) The protein expression of FDFT1 in CRC cells treated with ATA (30 μM) for 24 h by Western blotting. The figure (**B**) shown was representative of three independent experiments. The data (**C**) were expressed as means ± SD (n = 3). *$P < 0.05$, **$P < 0.01$, and ***$P < 0.001$ vs Ctrl (0 μM).

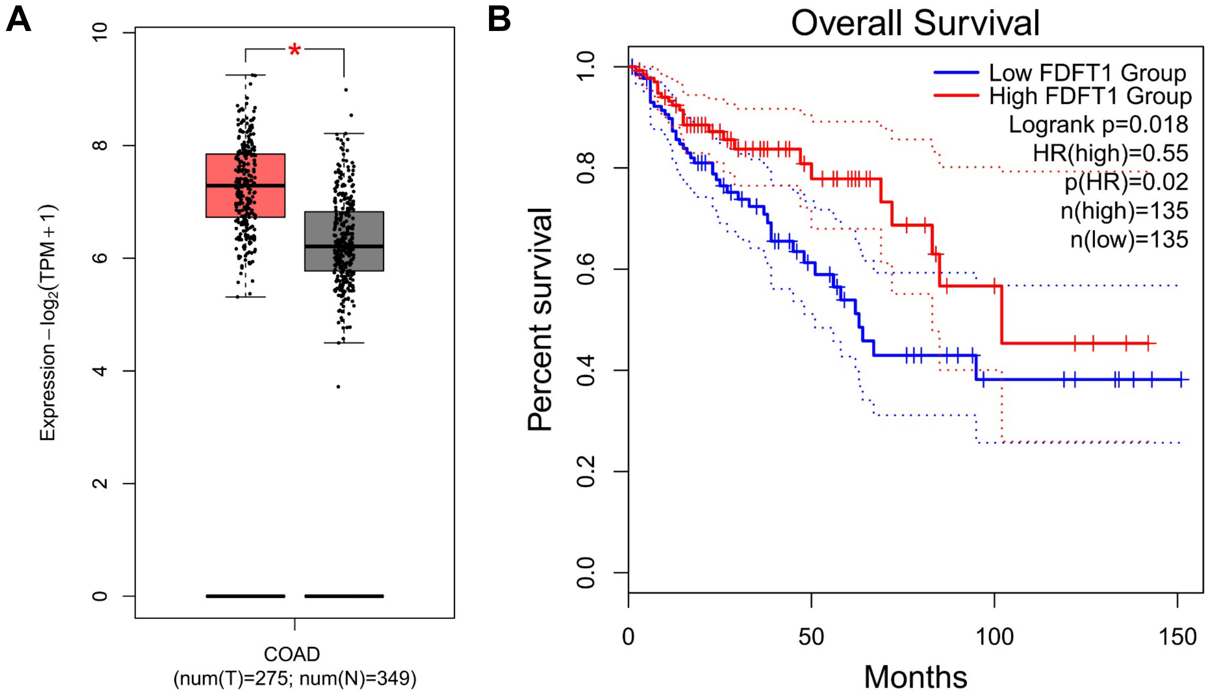

Figure S6. Box plots of mRNA expression in colon carcinoma tissues (**A**) and Kaplan-Meier survival plot (**B**) for FDFT1 in a population of colon cancer patients from GEPIA database (http://gepia.cancer-pku.cn/) matching TCGA normal and GTEx data. T: tumor; N: normal. The data are expressed as means ± SD. $^{*}P < 0.05$ *vs* Normal.

Table S1. The in vitro cytotoxic effect of ATA and analogue compounds on kinds of human cancer and normal cell lines.

No.	Cell line	Tissue information	IC$_{50}$ (μg/ml)						
			Cisplatin	Oxaliplatin	*β*-Elemene	23-HA	ATA	OA	UA
1	J82	Bladder cancer	0.40±0.19	6.47±0.69	16.87±0.52	13.38±0.35	10.59±0.94	> 100	6.26±0.36
2	SW1710	Bladder cancer	5.49±0.26	30.53±1.34	30.65±2.40	14.27±0.20	12.13±0.35	> 100	4.42±0.04
3	T24	Bladder cancer	1.47±0.14	13.32±1.22	27.62±0.88	12.33±1.06	7.44±2.30	65.13±26.14	4.62±0.08
4	UM-UC-3	Bladder cancer	0.89±0.31	4.61	17.72±0.02	8.44±1.30	4.81±0.75	> 100	5.29±0.54
5	MCF-7	Breast cancer	7.81±2.97	1.71±0.05	26.05±1.34	12.14±3.41	6.16±1.28	44.38±1.52	8.34±1.83
6	T47D	Breast cancer	26.16±9.11	18.18±1.77	37.79±4.62	15.80±2.32	13.43±4.93	> 100	5.24±0.11
7	DLD-1	Colon cancer	3.95±0.29	3.70±1.16	35.40±2.84	6.81±0.28	4.40±0.91	40.84±2.03	6.02±0.94
8	HCT15	Colon cancer	5.50±0.14	4.12±0.45	35.11±0.62	4.88±0.47	3.91±1.15	39.91±2.42	5.42±0.30
9	HepG2	Liver cancer	14.92±1.27	6.27±1.32	37.62±1.10	13.84±0.33	11.37±0.27	> 100	8.01±2.00
10	NCI-H460	Lung cancer	0.55±0.09	0.94±0.03	38.98±0.80	8.00±0.83	4.33±0.07	51.13±1.23	5.88±0.29
11	Caov-3	Ovary cancer	3.38±0.52	24.50±0.54	24.03±0.57	20.02±2.19	12.28±0.81	> 100	5.24±0.35
12	LNCap clone FGC	Prostate cancer	4.87±0.15	0.25	48.48	9.55±0.55	3.12±0.10	> 100	3.38±0.70
13	Caki-1	Renal cancer	2.49±0.56	2.63±0.88	32.83±0.59	12.96±1.46	8.53±0.45	> 100	5.43±0.37
14	HUVEC	Human umbilical vein endothelial cells	10.99±3.85	6.50±1.88	14.68±5.10	12.35±0.47	10.81±0.38	20.54±1.46	3.67±0.29
15	MRC-5	Lung fibroblast	2.19±1.84	1.34	17.90±0.71	14.20±1.86	9.53±0.52	> 100	6.38±2.89

Table S2. TOP20 hub genes predicted by 8 algorithms in cytoHubba plug-in of Cytoscape.

MNC	Degree	EPC	BottleNeck	Closeness	Radiality	Betweenness	Stress
TP53	TP53	TP53	TP53	TP53	TP53	TP53	TP53
VEGFA	VEGFA	JUN	VEGFA	JUN	JUN	JUN	JUN
JUN	JUN	VEGFA	JUN	VEGFA	VEGFA	HSP90AA1	HSP90AA1
HSP90AA1	HSP90AA1	HSP90AA1	PRKACB	HSP90AA1	HSP90AA1	VEGFA	VEGFA
CDH1	CDH1	CDH1	HSP90AA1	CDH1	CDH1	CDH1	CDH1
CXCL8	CXCL8	CXCL8	TTN	CXCL8	FYN	FYN	BDNF
BDNF	BDNF	MMP9	MUC4	MMP9	MMP9	BDNF	CXCL8
HSPA5	HSPA5	HSPA5	NRP1	FYN	CXCL8	PRKACB	FYN
MMP9	MMP9	CCNA2	HSPA8	BDNF	HSPA5	CXCL8	PRKACB
FYN	FYN	BDNF	THBS1	HSPA5	BDNF	HSPA8	HSPA5
HSPA8	FOXP3	FOXP3	PTGS2	HSPA8	HSPA8	HSPA5	HSPA8
FOXP3	HSPA8	PTGS2	NCAM1	FOXP3	PRKACB	HMOX1	HMOX1
CCNA2	CCNA2	CDKN1A	SYNE1	PRKACB	HMOX1	THBS1	MMP9
HMOX1	PTGS2	SERPINE1	ATF4	PTGS2	PTGS2	MMP9	FOXP3
PTGS2	HMOX1	HMOX1	HMOX1	HMOX1	FOXP3	PTGS2	THBS1
BMP4	BMP4	DDIT3	CDH1	CCNA2	SNCA	TYMS	PTGS2
SQSTM1	TYMS	ATF3	MMP9	SNCA	SQSTM1	FOXP3	CCNA2
DDIT3	SQSTM1	HSPA8	JAK1	SQSTM1	CCNA2	PHGDH	TYMS
PCNA	PCNA	EIF2AK3	KPNA2	TNFRSF1A	EIF2AK3	FASN	PHGDH
TYMS	DDIT3	PCNA	FYN	EIF2AK3	HSPA1A	STX1A	SQSTM1

Table S3. Key transcriptional factors of hub genes.

TF	Description	*P* value	Genes
STAT3	signal transducer and activator of transcription 3	7.30E-11	PTGS2, MMP9, VEGFA, CDH1, HMOX1, TP53
HDAC1	histone deacetylase 1	2.96E-10	MMP9, CDH1, PTGS2, TP53, HSPA5
TFAP2A	transcription factor AP-2 alpha	2.96E-10	VEGFA, MMP9, PTGS2, HMOX1, CDH1
STAT1	signal transducer and activator of transcription 1, 91kDa	7.00E-10	TP53, MMP9, PTGS2, HSP90AA1, HMOX1
AR	androgen receptor	1.18E-09	HMOX1, CDH1, JUN, PTGS2, VEGFA
KLF4	Kruppel-like factor 4	5.65E-09	VEGFA, HSPA8, TP53, CDH1
RELA	v-rel reticuloendotheliosis viral oncogene homolog A	6.76E-09	MMP9, VEGFA, PTGS2, TP53, CDH1, HMOX1
NFKB1	nuclear factor of kappa light polypeptide gene enhancer in B-cells 1	7.03E-09	CDH1, VEGFA, MMP9, PTGS2, TP53, HMOX1
JUN	jun proto-oncogene	1.27E-08	MMP9, PTGS2, JUN, VEGFA, TP53
PPARG	peroxisome proliferator-activated receptor gamma	4.42E-08	HMOX1, PTGS2, MMP9, TP53
ESR1	estrogen receptor 1	7.84E-08	TP53, VEGFA, JUN, CDH1
HMGA1	high mobility group AT-hook 1	9.97E-08	PTGS2, CDH1, TP53
HDAC3	histone deacetylase 3	2.96E-07	JUN, VEGFA, CDH1
HDAC4	histone deacetylase 4	2.96E-07	VEGFA, PTGS2, JUN
DNMT1	DNA (cytosine-5-)-methyltransferase 1	6.56E-07	CDH1, VEGFA, TP53
SMAD3	SMAD family member 3	6.56E-07	VEGFA, MMP9, JUN
ATF4	activating transcription factor 4	9.55E-07	PTGS2, HSPA5, VEGFA
PPARA	peroxisome proliferator-activated receptor alpha	1.33E-06	HMOX1, MMP9, PTGS2
SIRT1	sirtuin 1	2.51E-06	CDH1, TP53, MMP9
ELF4	E74-like factor 4	3.09E-06	MMP9, TP53
EP300	E1A binding protein p300	4.02E-06	VEGFA, PTGS2, MMP9
FOS	FBJ murine osteosarcoma viral oncogene homolog	4.24E-06	MMP9, TP53, PTGS2
WT1	Wilms tumor 1	4.24E-06	JUN, VEGFA, CDH1
FOXQ1	forkhead box Q1	4.63E-06	VEGFA, CDH1
HDGF	hepatoma-derived growth factor	4.63E-06	CDH1, VEGFA
IKBKB	inhibitor of kappa light polypeptide gene enhancer in B-cells, kinase beta	4.63E-06	TP53, MMP9
KLF8	Kruppel-like factor 8	8.63E-06	MMP9, CDH1
MEF2C	myocyte enhancer factor 2C	8.63E-06	JUN, VEGFA
ELF3	E74-like factor 3	1.11E-05	MMP9, PTGS2
HIPK2	homeodomain interacting protein kinase 2	1.11E-05	TP53, VEGFA
ETS1	v-ets erythroblastosis virus E26 oncogene homolog 1 (avian)	1.14E-05	HMOX1, TP53, MMP9
ABL1	c-abl oncogene 1	1.39E-05	TP53, JUN
SPDEF	SAM pointed domain containing ets transcription factor	1.39E-05	MMP9, CDH1
EGR1	early growth response 1	1.57E-05	VEGFA, TP53, PTGS2
CREB1	cAMP responsive element binding protein 1	1.68E-05	HMOX1, PTGS2, JUN
SNAI2	snail homolog 2	2.80E-05	CDH1, MMP9
ING4	inhibitor of growth family, member 4	3.23E-05	PTGS2, TP53
FOXM1	forkhead box M1	4.18E-05	CDH1, VEGFA

Table S3. Key transcriptional factors of hub genes (continued).

TF	Description	*P* value	Genes
GLI2	GLI family zinc finger 2	4.70E-05	JUN, CDH1
HDAC9	histone deacetylase 9	4.70E-05	TP53, HSPA5
ESR2	estrogen receptor 2	5.25E-05	JUN, VEGFA
XBP1	X-box binding protein 1	5.25E-05	VEGFA, HSPA5
E2F1	E2F transcription factor 1	5.54E-05	TP53, HSPA5, VEGFA
ARNT	aryl hydrocarbon receptor nuclear translocator	5.84E-05	VEGFA, JUN
KLF6	Kruppel-like factor 6	5.84E-05	CDH1, MMP9
VHL	von Hippel-Lindau tumor suppressor, E3 ubiquitin protein ligase	5.84E-05	TP53, VEGFA
CTNNB1	catenin, beta 1, 88kDa	7.09E-05	PTGS2, JUN
MTA1	metastasis associated 1	8.47E-05	MMP9, CDH1
CREBBP	CREB binding protein	9.20E-05	TP53, PTGS2
PARP1	poly (ADP-ribose) polymerase 1	9.20E-05	JUN, CDH1
PGR	progesterone receptor	9.20E-05	PTGS2, VEGFA
TCF4	transcription factor 4	9.20E-05	JUN, VEGFA
HDAC2	histone deacetylase 2	0.000	CDH1, VEGFA
RB1	retinoblastoma 1	0.000	VEGFA, CDH1
ATF2	activating transcription factor 2	0.000	PTGS2, JUN
ETS2	v-ets erythroblastosis virus E26 oncogene homolog 2 (avian)	0.000	TP53, MMP9
EZH2	enhancer of zeste homolog 2 (Drosophila)	0.000	CDH1, TP53
RUNX1	runt-related transcription factor 1	0.000	VEGFA, JUN
USF2	upstream transcription factor 2, c-fos interacting	0.000	PTGS2, HMOX1
IRF1	interferon regulatory factor 1	0.000	TP53, MMP9
BRCA1	breast cancer 1, early onset	0.000	HSPA5, VEGFA
USF1	upstream transcription factor 1	0.001	PTGS2, HMOX1
HIF1A	hypoxia inducible factor 1, alpha subunit	0.001	VEGFA, CDH1
YY1	YY1 transcription factor	0.001	TP53, HSPA5
MYC	v-myc myelocytomatosis viral oncogene homolog (avian)	0.001	VEGFA, TP53
SP1	Sp1 transcription factor	0.002	VEGFA, PTGS2, MMP9
TP53	tumor protein p53	0.004	TP53, VEGFA

Table S4. The predicted targets of 3β-hydroxy-12-oleanen-27-oic acid (ATA), oleanolic acid (OA), and ursolic acid (UA).

Compound	Predicted targets
**ATA**	CD81, PTPN2, PTPN1, HSD11B2, ESR2, NR1H3, CYP19A1, SERPINA6, SHBG, G6PD, CYP51A1, PTPN6, CDC25A, TERT, CES2, PREP, FABP1, HMGCR, CYP17A1, NOS2, SCD, POLB, RORC, PDE4D, PTPRF, PLA2G1B, ACP1, PPARG, HSD11B1, CDC25B, PTPN11, NPC1L1, RORA, PPARA, PTGIR, PTGS2, PTGES, AR, SIGMAR1, PTGS1, AKR1B10, FFAR1, PPARD, ESR1, SLC6A4, CYP2C19, FABP4, FABP3, FABP5, IDO1, SREBF2, CTSD, FAAH, SLC6A3, ADORA3, MAPK3, SRD5A2, BCHE, PTGER2, TOP1, TOP2A, NR3C1, PTGER1, NR3C2, FDFT1, ITGAL, TNF, PTGER3, SLC22A6, PRKCH
**OA**	POLB, PTPN1, PTPN2, PLA2G1B, CDC25B, AKR1B10, RORC, PTPRF, HSD11B1, ACP1, PDE4D, CD81, PTGES, CES2, PTPN6, NOS2, FABP1, AR, PPARG, PREP, SCD, HSD11B2, PRKCH, PTPN11, FNTA FNTB, TERT, PTGS2, CYP19A1, FABP4, PPARA, PPARD, SERPINA6, SHBG, G6PD, CYP51A1, CDC25A, LTB4R, PTGS1, FABP3, FABP5, ESR2, FAAH, NPC1L1, SIGMAR1, CYP17A1, PTGER2, ADORA3, MAPK3, NR1H3, GPBAR1, NR3C1, SAE1 UBA2, SLC6A4, CYP2C19, TOP1, HMGCR, TLR9, NR1H4, ALOX5, PGR, PTGER4, ALOX5AP, CHRM2, SLC6A2, NR3C2, GRIK1, GRIK2, ESR1, FFAR1, SRD5A2, RORA, BCHE, PTGIR, NR1I3, CES1, MDM2, BACE1, SLC6A3
**UA**	CES2, PTPN1, PTPN2, AKR1B10, PTGES, HSD11B1, PRKCH, HSD11B2, PTPN11, POLB, PLA2G1B, CDC25B, RORC, PTPRF, ACP1, PDE4D, PTPN6, FABP1, PPARG, AR, NR1H3, SCD, PREP, CDC25A, SERPINA6, SHBG, G6PD, CYP51A1, TOP1, CD81, CYP19A1, NPC1L1, SIGMAR1, CYP17A1, FABP4, PPARA, TERT, PPARD, SAE1 UBA2, NOS2, NR3C1, ADORA3, MAPK3, FABP3, FABP5, HMGCR, PTGS1, PGR, FNTA FNTB, FAAH, BCHE, BACE1, RORA, ALOX5, ESR2, PTGER2, TOP2A, IDO1, SLC6A3, PTGER1, CHRM2, SLC6A2, SLC6A4, CYP2C19, FFAR1, SRD5A2, PTGS2, CES1, PTGER4, NR1I3, LTB4R, SREBF2, ESR1, NR1H4, TRPV1, GPBAR1, ALOX5AP, CNR1, ACHE, NR1I2, HSD17B3, SLC22A6, CDC25C, NR3C2, HSD17B2, PTGIR, EPHA2

Table S5. Primer sequences used for real-time qPCR.

Gene name	Forward (5′-3′)	Reverse (5′-3′)	Product (bp)
CHAC1	GAAACGACGGCGACCCTCAAG	TGCCCCGATGGAAGGTGTCTC	147
DDIT3	GAGAATGAAAGGAAAGTGGCAC	ATTCACCATTCGGTCAATCAGA	120
DDIT4	GATGCCTAGCCAGTTGGTAAG	CTAAACAGCCCCTGGATCTTG	228
DKK1	TACCAGACCATTGACAACTACC	TCCATTTTTGCAGTAATTCCCG	189
ID2	GAAGGTGAGCAAGATGGAAATC	ACTCAGAAGGGAATTCAGAAGC	200
KITLG	GTGGCAAATCTTCCAAAAGACT	CCATCTCGCTTATCCAACAATG	91
FDFT1	GGCAGTACCTGACCACTCTC	TCCTAAAGGTCCCAGCCACA	200
GAPDH	CATGGGTGTGAACCATGAGAA	GGTCATGAGTCCTTCCACGAT	133
